# The tissue and circulating cell‐free DNA‐derived genetic landscape of premalignant colorectal lesions and its application for early diagnosis of colorectal cancer

**DOI:** 10.1002/mco2.70011

**Published:** 2024-11-14

**Authors:** Qingjian Chen, Yu‐Hong Xu, Shiyang Kang, WuHao Lin, Linna Luo, Luping Yang, Qi‐Hua Zhang, Pan Yang, Jia‐Qian Huang, Xiaoni Zhang, Jing Zhang, Qi Zhao, Rui‐Hua Xu, Hui‐Yan Luo

**Affiliations:** ^1^ Department of Medical Oncology Sun Yat Sen University Cancer Center State Key Laboratory of Oncology in South China Collaborative Innovation Center for Cancer Medicine Sun Yat‐sen University Guangzhou Guangdong China; ^2^ Research Unit of Precision Diagnosis and Treatment for Gastrointestinal Cancer Chinese Academy of Medical Sciences Guangzhou Guangdong China; ^3^ Department of Oncology State Key Laboratory of Systems Medicine for Cancer Shanghai General Hospital Shanghai Jiao Tong University School of Medicine Shanghai Shanghai China; ^4^ Department of Anaesthesiology Sun Yat Sen University Cancer Center State Key Laboratory of Oncology in South China Collaborative Innovation Center for Cancer Medicine Sun Yat‐sen University Guangzhou Guangdong China; ^5^ Department of Endoscopy Sun Yat Sen University Cancer Center State Key Laboratory of Oncology in South China Collaborative Innovation Center for Cancer Medicine Sun Yat‐sen University Guangzhou Guangdong China; ^6^ HaploX Biotechnology Shenzhen China

**Keywords:** circulating cell‐free DNA, colorectal adenomas, colorectal cancer, premalignant lesions

## Abstract

Colorectal adenomas (CRAs) represent precancerous lesions that precede the development of colorectal cancer (CRC). Regular monitoring of CRAs can hinder the progression into carcinoma. To explore the utility of tissue DNA and circulating cell‐free DNA (cfDNA) in early diagnosis of CRC, we retrospectively sequenced paired tissue and plasma samples from 85 patients with conventional CRAs. The genetic alterations identified were compared with those from 78 stage‐I CRC patients (CRC‐I) in the ChangKang project. Within the CRA cohort, we pinpointed 12 genes, notably *APC*, *KRAS*, and *SOX9*, that exhibited significant mutated rates in tissue. Patients harboring *KMT2C* and *KMT2D* mutations displayed persistent polyps. By comparing with the mutational profiles of metastatic CRC plasma samples, we found that *ZNF717* was exclusively mutated in CRAs, while *KMT2C* and *KMT2D* mutations were detected in both CRA and CRC. The presence of cfDNA mutations in plasma was validated through polymerase chain reaction, enhancing the feasibility of using cfDNA mutations for early CRC screening. Compared with CRC‐I, CRAs exhibited a reduced frequency of *TP53* and *PIK3CA* somatic mutations and underwent non‐neutral evolution more often. We established a random forest model based on 15 characteristic genes to distinguish CRA and CRC, achieving an area under the curve of 0.89. Through this endeavor, we identified two novel genes, *CNTNAP5* and *GATA6*, implicated in CRC carcinogenesis. Overall, our findings reveal convincing biomarkers markers for detecting CRAs with a propensity for CRC development, highlighting the importance of early genetic screening in CRC prevention.

## Graphical abstract

In this paper, to explore the pathogenesis of the progression from precancerous lesions to adenocarcinoma and propose potentially feasible approaches for the early detection of CRC, we retrospectively sequenced paired tissue and plasma samples from 85 patients with conventional colorectal adenomas (CRAs). The identified genetic alterations were compared with those from 78 CRC stage‐I patients (CRC‐I) from the ChangKang Project

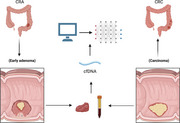

## 1 INTRODUCTION

CRC is the third most prevalent cancer and the second leading cause of cancer‐related death worldwide, accounting for 10% of all cancer incidence and 9.4% of mortality rates.^1^ Despite encouraging advancements in early detection methods, most patients are still diagnosed with advanced CRC at the initial consultation, highlighting the critical need for the development of more effective early detection approaches to improve the overall prognosis for CRC patients. CRAs, being the precancerous lesions of CRC, underscore the importance of their management for the early prevention of potentially advanced CRC.

The tumorigenesis of CRC involves numerous genomic alterations.^2^ Large‐scale sequencing projects, exemplified by the Cancer Genome Atlas (TCGA) and other consortia, have revealed the molecular underpinnings of CRC.^3^, ^4^ Recent studies have investigated the genomic landscapes of premalignant CRAs and the evolutionary transition from premalignant to malignant in colorectal tissues.^5^, ^6^ There are two major types of CRAs: conventional adenomas and sessile serrated adenomas (SSAs).^7^ In conventional adenomas, CRC arises from tubular adenomas (TAs), villous adenomas (VAs), and mixed tubulovillous adenomas (TVAs), often via activation of the WNT and KRAS pathways.^8^ Conversely, SSAs are characterized by microsatellite instability (MSI), epigenetic disruptions in MLH1, the CpG island methylator phenotype, and BRAF activation, particularly V600E mutations.^8^ SSAs constitute 10−20% of polyps and are more commonly found in the proximal than in the distal colon. The precancerous lesions share many molecular traits with their malignant counterparts.^9^ By comparing genetic profiles, CRAs are categorized into two consensus molecular subtype (CMS) subgroups: CMS1‐like serrated adenomas and CMS2‐like canonical adenomas.^10^, ^11^ CMS2‐like adenomas exhibit enriched WNT and MYC signaling, while CMS1‐like adenomas are characterized by MSI and prominent immune infiltration. However, the identification of molecular biomarkers for high‐risk adenomas remains contentious. It is estimated that only about 5% of advanced CRAs eventually evolve into CRC.^7^ Prior research has classified adenomas into low‐ and high‐risk categories, primarily based on histology, which is thought to correlate with an elevated CRC risk. Early detection and removal of these adenomas can significantly mitigate CRC risk. However, aside from colonoscopy, there is a dearth of efficient and precise methods to identify high‐risk lesions. Colonoscopy stands as the premier screening tool due to its ability to visually inspect the entire colon and rectum and promptly biopsy or remove abnormalities. For instance, patients with 3−10 TAs, more than 10 adenomas, one or more TAs ≥10 mm in size, or one or more VA are recommended to undergo regular surveillance every 3 years.^7^ Nonetheless, the association between histology and malignancy risk remains a matter of debate.^2^, ^12^ Balancing potential overtreatment of low‐risk adenomas with early detection of high‐risk adenomas is paramount in cancer prevention.

For most CRC patients, early detection of tumors is critical for improving patient survival. Colonoscopy, while considered the gold standard for screening early‐stage CRC, possesses drawbacks including time‐consuming procedures, high cost, and invasiveness. CfDNA) referring to DNA fragments released into the blood plasma by cells undergoing apoptosis, necrosis, and other physiological processes, has emerged as a key player in disease diagnosis and monitoring through liquid biopsy techniques.^13^, ^14^ Our previous works have demonstrated that cfDNA‐based models can improve diagnosis, treatment, and prognosis of various tumors, notably hepatocellular carcinoma and CRC.^15^, ^16^, ^17^, ^18^ The genomic profile of cfDNA mutations is an effective biomarker for metastatic CRC, enabling earlier detection of tumor recurrence than the traditional computed tomography methods.^18^ However, the application of liquid biopsy in precancerous lesions remains controversial. Previous studies have established cfDNA‐based models to distinguish patients with and without advanced adenoma (AA), leveraging either 11 methylation biomarkers or low‐pass whole genome sequencing.^19^, ^20^ In contrast, the release of DNA fragments from precancerous lesions into the plasma is still debated. For instance, cancer‐related mutations (KRAS or BRAF) in CRAs failed to be detected in plasma samples using droplet digital PCR,^21^, ^22^ Moreover, a model relying on six methylation markers cannot identify CRAs unless the median tumor size exceeds 1.5 cm.^23^ Therefore, the feasibility of applying cfDNA analysis for precancerous lesions has yet to be fully established.

In a recent study, we launched the ChangKang (Healthy Bowel) Project, which aims to establish a comprehensive genomic and clinical database of 1000 CRC patients from the Chinese population.^4^ This study continues the follow‐up work of the ChangKang Project, focusing on the early screening and diagnosis of CRC. Our primary objective is to delineate the genomic landscapes of CRAs and to test the feasibility of detecting gene mutations within these precancerous lesions. To this end, we collected paired tissue samples and cfDNA samples from 85 Chinese patients with CRAs at varying stages. The in‐depth analysis of the comprehensive genomic landscapes of CRAs presents opportunities for the identification of molecular biomarkers that can pinpoint high‐risk adenomas. Additionally, we developed a random forest model based on 15 genes to distinguish CRA from CRC. Finally, we evaluated the potential of cfDNA for the early detection of CRAs. The findings of this study offer insights into the pathogenesis of the progression from precancerous lesions to adenocarcinoma and propose potentially feasible approaches for the early detection of CRC.

## 2 RESULTS

### 2.1 CRA patients’ characteristics and landscapes of somatic mutations in tumor tissue samples

**FIGURE 1:**
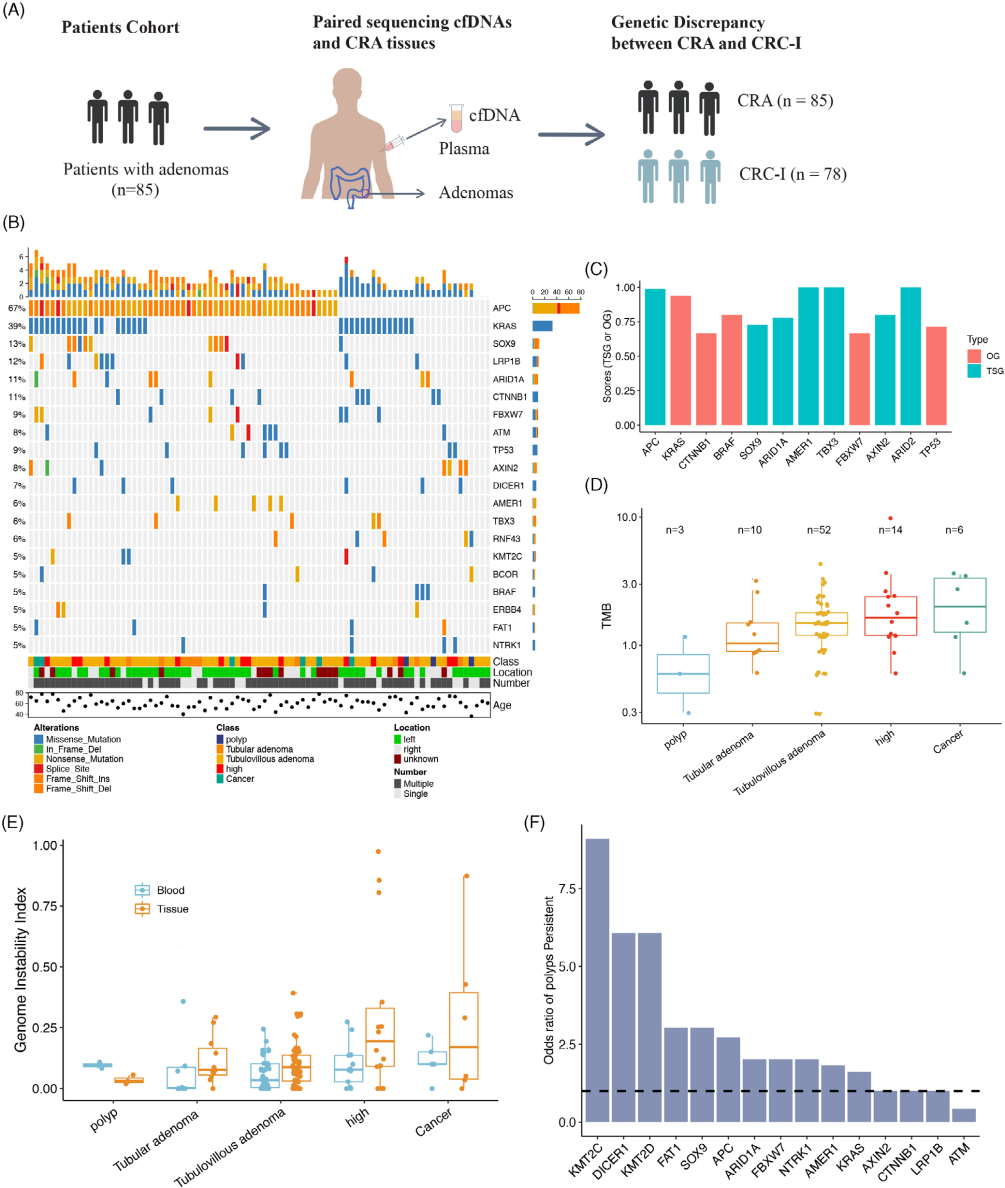
Study overview and mutation characteristics of CRA. (A) Study design schematic. The patients with colorectal adenomas were selected based on pathological evaluation. The adenomas tissues, the plasma cfDNA, and leukocyte (as normal tissue) were sent for target panel sequencing. The genetic discrepancies between CRA and CRC‐I were investigated to reveal the genomic features and cancer evolutions from precancerous lesions into carcinomas. (B) The genetic landscape of CRA tissue samples. (C) The oncogenic scores and TSG scores are defined by SomInaClust for each gene. (D) The boxplot showing the TMB of CRA tissues from patients with polyps (*n* = 3), tubular adenoma (*n* = 10), tubulovillous adenoma (*n* = 52), high‐risk adenoma (*n* = 14), and cancer (*n* = 6). (E) The genome instability index of CRA tissues and plasma samples from patients with different stages of CRAs. (F) The risk genes for persistent polyps by long‐term follow‐up.

To investigate the mutational profiles of CRAs, we retrospectively collected tumor tissue and cfDNA from 85 patients with conventional CRAs (Figure 1A). Both the tumor tissue and cfDNA were sequenced using a cancer gene‐targeted panel that covers 451 genes. The patient cohort included three (3.53%) polyps, 10 (11.76%) TAs, 52 (61.18%) TVAs, 14 (16.47%) high‐risk adenomas, and six (7.06%) adenomas mixed with early‐stage cancer. The overall characteristics of patients are presented in Table S1. The mean age of patients with CRA was 60.88 years (±9.23), with women comprising 41.18% of the total patient population. A higher percentage of adenomas were located on the left side compared with the right side (56.47 vs. 25.88%), and most patients had multiple adenomas rather than a single one (80.00 vs. 20.00%).

We identified 477 nonsynonymous and 178 synonymous mutations spanning 162 genes in CRA tissues. In the CRA tissue samples, the genes APC, KRAS, SOX9, LRP1B, ARID1A, and CTNNB1 were mutated in over 10% of patients (Figure 1B). Utilizing SomInaClust to analyze the mutation patterns of oncogenes and tumor suppressor genes (TSGs),^24^ we identified five oncogenes and seven TSGs in our cohort with a *Q*‐value < 0.01 (Figure 1C). All these genes were classified as CRC driver genes in IntOGen.^25^ Consistent with previous CRA studies,^5^ APC and KRAS emerged as the most frequently mutated genes in the classical CRC pathway, with mutation rates of 67 and 39%, respectively. Notably, APC mutations were predominantly nonsense and frameshift mutations, aligning with its roles as a TSG in CRC.^5^ KRAS, functioning as an oncogene, presented exclusively as missense mutations, particularly at its oncogenic sites (*KRAS* G12). The oncogene *BRAF*, commonly associated with SSAs, was found in four patients (including three TVAs and one TA) and was exclusively comutated with KRAS, corroborating previous findings on conventional adenomas.^5^ Surprisingly, we identified *TP53* as an oncogene in CRA, as all mutations were missense, which contradicts its known role as a TSG in tumorigenesis. This finding is supported by previous research suggesting that *TP53* may have oncogenic properties in several types of tumors.^26^

Next, we analyzed the genomic burden across different malignancy stages of CRA tissue samples. The tumor mutation burden (TMB), defined as the number of somatic mutations per megabase (Mb), was estimated, revealing a median TMB of 1.50 (ranging from 0.30 to 9.88). The level of TMB in CRAs was slightly lower than that observed in CRC patients from the Chinese ChangKang Project (median TMB of 1.74) but was significantly lower compared with the TMB of the TCGA CRC cohort (median TMB of 2.28). Additionally, we investigated the association between TMB and various clinical features. The TMB was comparable across different adenomas’ locations, as well as among patient age and gender groups. Interestingly, patients with multiple adenomas had a slightly lower TMB compared with those with a single adenoma (Wilcoxon test, *p* = 0.0652), suggesting different underlying malignant mechanisms between these two groups. We also examined TMB levels at different CRA stages. Overall, TMB increased progressively from polyps to cancers, indicating that the accumulation of somatic mutations could elevate the malignancy levels in CRAs (Figure 1D). This positive association between TMB levels and malignancy in precancerous lesions has been observed in many cancers, including CRC ^27^ and lung cancer.^28^

### 2.2 Copy number variants in CRA tissues and plasma samples

In our study, we conducted a genome‐wide analysis of allele‐specific copy number variations (CNVs) in both tissue samples and matched plasma samples to uncover significant genomic alterations across different stages of CRAs. To accurately determine tumor purity, tumor ploidy, and the genome‐wide allele‐specific CNVs, we employed the FACETS algorithm with a low‐sensitivity setting (cval = 150).^29^ Our analysis showed that all plasma samples were diploid, while tissue samples exhibited notable CNVs, with 20 samples (20 out of 85, 23.5%) displaying a ploidy greater than 2. The malignant levels of CNVs were evaluated by using the genome instability index (GII), which quantifies the proportion of the genome affected by copy number loss, copy number gain and copy‐neutral loss of heterozygosity.^30^ Notably, the average GII in tissue samples was significantly higher than in plasma samples (14.6 versus 7.7%, Mann–Whitney test *p* = 0.01). Furthermore, we observed a positive correlation between the GII scores and the levels of malignancy in both CRA tissues and plasma samples (Figure 1E). These results suggest that the spectrum of CNVs can be a powerful marker for gauging the malignancy levels of CRA in both tissue and plasma samples.^19^, ^20^

### 2.3 Discovery of risk genes for persistent polyps by long‐term follow‐up

To further understand the relationship between patient's condition and the genetic landscapes, we conducted an information backtrack. However, due to the challenges of regular re‐examinations for CRA patients, as of May 2024, we have only obtained the follow‐up status of 44 patients. A total of 19 and 23 patients were at polyp‐free or polyps’ persistent status after complete or incomplete removal of polyps, respectively. Only two patients were found to be cancerous after 3 years and started anticancer therapy in 2021 (Table S2). To estimate the genes that contribute to the persistence of polyps, we use the odds ratio of Fisher's exact test:

$$R_{i}=\frac{m_{i}/n_{i}}{M/N}$$

where $m_{i}$ and $n_{i}$ are the number of polyp‐persistent and polyp‐free patients for gene i, respectively. The M and N are the total number of polyp‐persistent and polyp‐free patients, respectively. This approach identified six genes that promote the polyp‐persistent status, including *KMT2C, DICER1, KMT2D, FAT1, SOX9*, and *APC* (Figure 1F). Patients with these mutated genes were recommended to undergo regular polyp surveillance. For the two patients with cancerous progression, the first patient was diagnosed as TVA mixing with carcinoma at the initial time and contained somatic mutations including *APC, KRAS*, and *ARID1A*. The other patient was TA status and had somatic mutations in *APC, KRAS*, and *KMT2C*.

### 2.4 Analysis of genomic alterations in plasma

**FIGURE 2:**
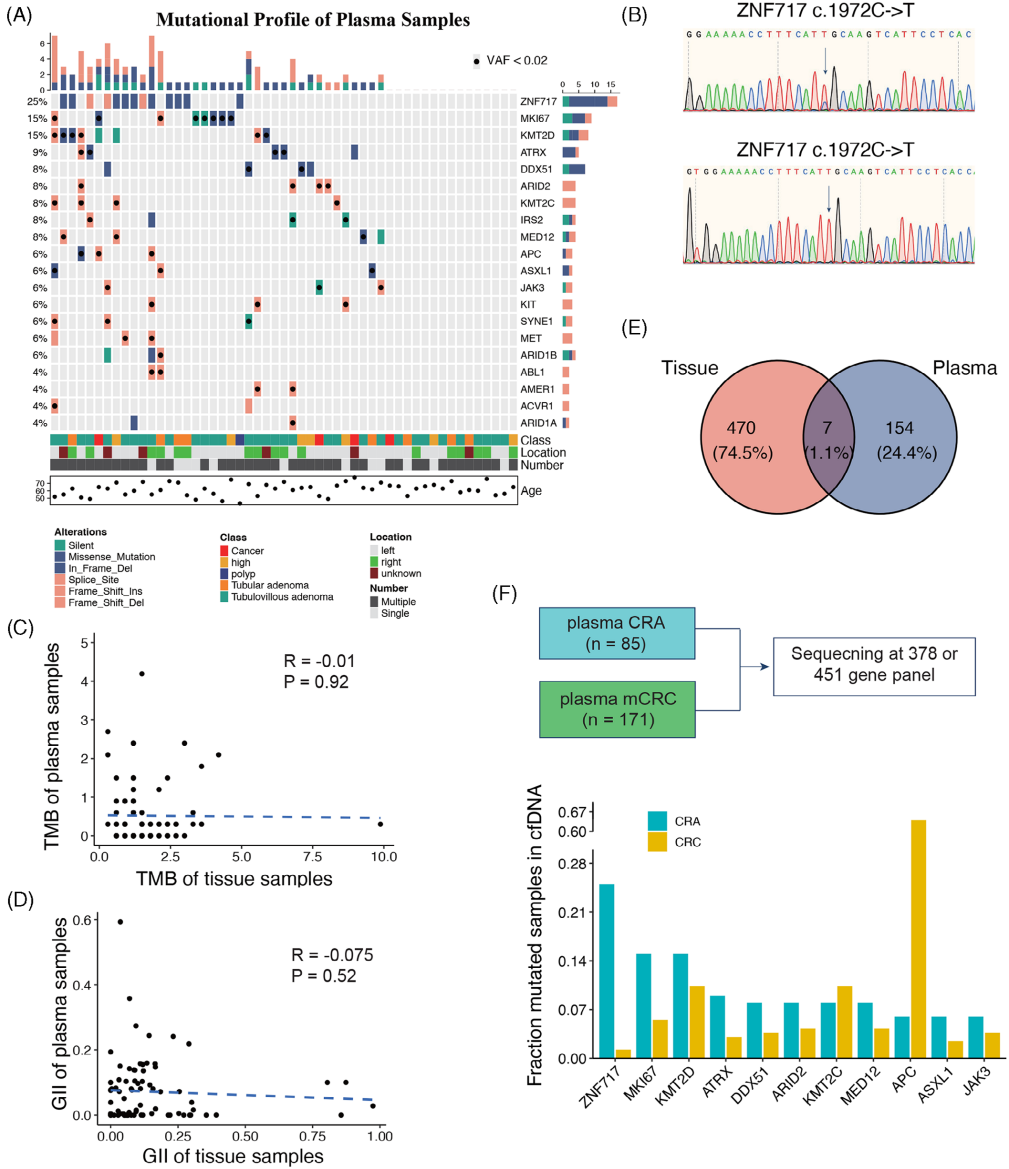
Comparison of genomic alterations between tissue and plasma samples. (A) The genetic landscape of CRA plasma samples. (B) The mutations of ZNF717 were detected by PCR and sequencing in patients’ plasma cfDNA. (C and D) The scatter plots show the relationship of TMB (C) and GII (D) between CRA tissues and plasma samples. (E) The number of overlapped somatic mutations between CRA tissues and plasma samples. (F) Genetic mutations in CRA and mCRC plasma samples.

In our study, we aimed to determine whether cancer‐specific mutations from precancerous lesions could be directly identified in plasma samples. We analyzed the somatic mutations in plasma samples and identified *ZNF717* as the most frequently mutated gene (25%) (Figure 2A). *ZNF717* is part of the zinc‐finger protein family, reported as a TSG by regulating the IL6‐STAT3 pathways in hepatocellular carcinoma.^31^ However, the mutant rate of *ZNF717* in the ChangKang cohort and the TCGA CRC cohort was only 1.38 and 1.5%, respectively. To further validate the mutation status of *ZNF717* in the plasma, we collected plasma samples from six patients who had previously been included in the analysis and detected the mutation of gene *ZNF717* by PCR and sequencing. Despite the disadvantages of the long storage time, unstable quality and low cfDNA content of the samples, we finally detected somatic mutation in two of the six samples, which were heterozygous and homozygous mutations, respectively (Figure 2B and Table S3). This result suggests that the gene mutation in plasma cfDNA can be detected by PCR and sequencing simply, which further verifies the application value of cfDNA in the early stage of cancer detection.

### 2.5 Tissue DNA and plasma cfDNA mutation profiles have their own uniqueness

We sought to determine if genomic alterations identified in CRA tissues could be mirrored in the matched cfDNA samples. To achieve this, we evaluated the levels of genomic concordance between tissue and plasma samples. Unfortunately, our analysis revealed that genomic alterations, including TMB and GII, did not show any significant correlation between tissue and plasma samples as evidenced by Spearman's correlation test (*p* = 0.92 for TMB and *p* = 0.52 for GII) (Figure 2C,D). These findings indicate that leveraging cfDNA detection for precancerous lesions poses more challenges than its application in carcinomas.

We further investigated whether somatic mutations detected in CRA tissues were also present in matched plasma samples. To ensure comprehensive detection, we imputed somatic mutations in plasma samples from raw binary alignment map (bam) files, minimizing the risk of overlooking mutations with low VAF. Among all somatic mutations identified in CRA tissues, only seven mutations across five patients were detectable in plasma samples, representing a detection rate of 5.9% (five out of 85 patients) (Figure 2E). The variant allele frequency (VAF) of these seven positive mutations ranged from 0.92 to 2.97%, highlighting the necessity of deep sequencing for such mutations. Further examination of the clinical features of these five patients found that all of them had multiple adenomas status (Table 1). Specifically, patient P5, with two identified cfDNA mutations, presented with multiple TAs measuring 4.5 × 5.0 cm, local mucosal muscle invasion, and an adjacent moderately to poorly differentiated adenocarcinoma measuring 3.5 × 4.0 cm. Patient P6, also with two cfDNA mutations had two VAs and one polyp in the ascending colon, alongside one VA and one TVA in the descending colon with the tumor size being 2.0 × 2.0 cm. The pathological assessments for the other patients were as follows: P7 and P8 each had two TVAs, and P9, a patient with postoperative rectal cancer had two TVAs, one poly and one laterally spreading tumor (LST) measuring 4.0 × 3.5 cm. Subsequently, we investigated whether these cfDNA‐positive patients exhibited specific driver mutations in their matched adenoma tissues. Notably, the presence of both *APC* and *KARS* mutations was slightly more common among the five cfDNA‐positive patients compared with the larger cohort (3/5 vs. 19/85, Binomial test *p* = 0.078). In summary, the risk factors associated with cfDNA‐positive adenomas included a mixture of CRA with adenocarcinoma, a large CRA size (exceeding 2 × 2 cm), and the copresence of *APC* and *KARS* mutations.

**TABLE 1:** Summary of mutations in plasma samples.

Genes	Variant classification	Protein change	Frequency^a^ (%)	VAF (%)	Sample IDs	Driver genes^b^	Pathology
*NOVA1*	Intron	*/*	1.2	2.87	P9	*KRAS*, two *APC*	Two TVAs, one polyp, one LST^c^ (4.0×3.5 cm)
*KRAS*	Missense mutation	p.G12V	2.4	0.92	P8	*KRAS*, *APC*	Two TVAs
*CCDC170*	Intron	*/*	1.2	9.38	P7	*SYNE1*	Two TVAs
*KIF1B*	Intron	*/*	1.2	2.97	P6	*AXIN2*	One TVA (2.0 × 2.0 cm), three VAs, and one polyp
*BAP1*	Intron	*/*	1.2	2.41			
*APC*	Frame shift del	p.G1394fs	5.8	0.94	P5	*KRAS*, two *APC*	One VA (4.5 × 5.0 cm), One cancer (3.4 × 4.0 cm)
*KRAS*	Missense mutation	p.G12V	2.4	1.57			

^a^ The overall mutation frequency in plasma samples.

^b^ Cancer driver genes in the matched tissue samples.

^c^ LST, laterally spreading tumor.

### 2.6 Comparison of cfDNA sequencing between CRA and CRC revealed the potential early prediction markers

To explore whether the markers detected in the CRA plasma can be revealed in the CRC plasma, we collected a metastatic CRC (mCRC) cohort from our previously published study (Figure 2F).^32^ The mCRC cohort contained 171 patients under 378‐gene panel sequencing. The gene panel used in the current study is the next version of the 378‐gene panel, containing more than 451 genes. By comparing the mutated frequency of plasma samples between CRA and mCRC, the gene *ZNF717* was exclusively mutated in the CRA, indicating this gene could serve as a marker for the prediction of CRA from patient plasma samples. Interestingly, the two genes, *KMT2C* and *KMT2D*, can be detected in both CRA and CRC (15 vs. 10% and 8 vs. 10%, respectively). The two genes were also the risk genes for polyp persistent status (Figure 1F). Detecting the two genes can not only monitor the polyp status, but also detect CRC in the very early stage before carcinoma.

### 2.7 Comparative analysis of mutation profiles in CRA and CRC‐I

**FIGURE 3:**
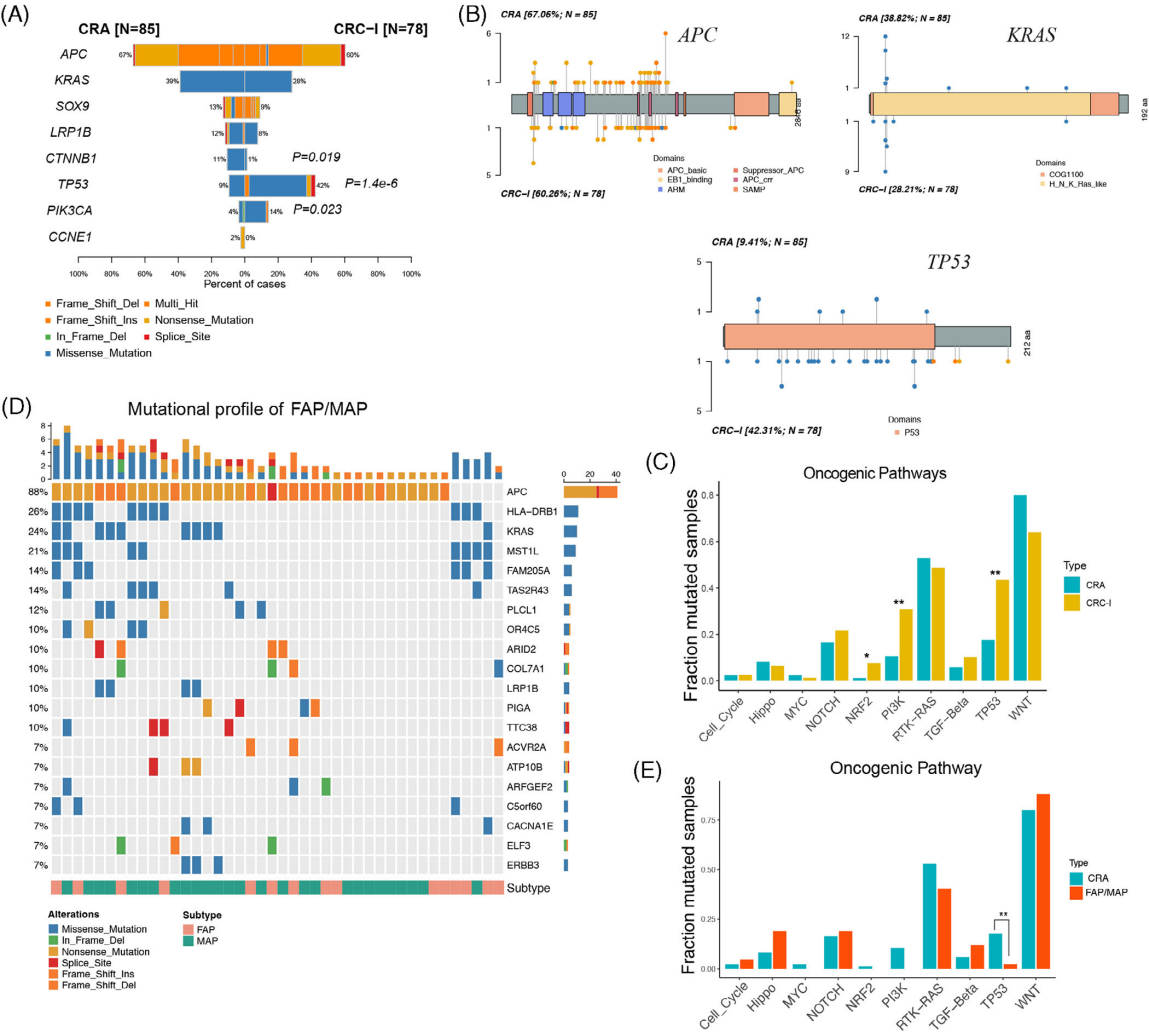
Characterization of genetic profiles of CRA and CRC‐I. (A) The frequently mutated genes in CRA and CRC‐I (CRA: *n* = 85; CRC‐I: *n* = 78). (B) Mutated loci are shown for APC, KRAS, and TP53 in CRA and CRC‐I. The proportion of mutated patients in CRA and CRC‐I and the protein domains in each gene are displayed. (C) Difference of oncogenic pathways in CRA and CRC‐I. The *p* values of Fisher's exact test was labeled. *: *p* < 0.05; **: *p* < 0.01. (D) The genetic landscape of FAP/MAP. (E) Difference of oncogenic pathways in CRA and CRC‐I.

To further elucidate the molecular transition from CRA to CRC, we analyzed CRC‐I patients (*n* = 78) from the ChangKang project. This substantial cohort of CRC‐I patients provided a unique opportunity to identify key genomic events marking the transition from CRA to CRC. Notably, we observed a significant increase in the mutation frequency of *TP53* from CRA to CRC‐I (9 vs. 42%, Fisher's exact *p* = 1.4 × 10^−6^), followed by *PIK3CA* (4 vs. 14%, *p* = 0.023) (Figure 3A). These alterations, which occur at higher frequencies in malignant conditions than their precancerous counterparts, were cancer‐driving aberrations. Conversely, genomic alterations of *KRAS*, *SOX9*, *LRP1B*, and *CCNE1*, had higher frequencies in CRA than in CRC‐I. The frequency of *CTNNB1* mutations showed a marked decline from 11% into 1% in CRC‐I (Fisher's exact test, *p *= 0.019). These genes, with higher mutation rates in premalignant lesions than in malignant ones, represented early events in the carcinogenic process and may require additional genetic changes to evolve into carcinomas.^33^

The mutation positions were crucial for the functions of genes during carcinogenesis. Despite in different stage, CRA and CRC‐I displayed similar mutation profiles for key genes like *APC*, *KRAS*, and *TP53* (Figure 3B). This suggests a shared functional mechanism in the early stages of colorectal tumorigenesis. Notably, most *KRAS* mutations in both CRA were found in well‐known functional hotspots, specifically *KRAS* G12. This was observed in 90.9% of *KRAS* in CRA (30 out of 33 mutations) and 86.4% in CRC‐I (19 out of 22 mutations), underscoring the critical role of these hotspots in cancer progression. For *TP53, s*omatic mutations were primarily missense types and occurred within the DNA‐binding domains in both CRA and CRC‐I. However, the frequency of *TP53* mutations dramatically increased alongside tumor grade progression (9.41% in CRA vs. 42.31% in CRC‐I). Despite a thorough investigation, no distinct *TP53* mutation hotspots were identified in either CRA or CRC‐I. This contrast with metastatic CRC (CRC‐IV), where specific *TP53* hotspots such as p.R43H, pR81X, p.R141H, and p.R64X were notably prevalent, indicating a pattern of mutational preference shifting in advanced stages of CRC.^4^

Additionally, we analyzed the involvement of cancer hallmark pathways between CRA and CRC‐I, focusing on the 10 well‐established oncogenic signaling pathways identified in the TCGA (Figure 3C).^34^  The WNT and RTK–RAS pathways were mainly mutated in CRA, with an adjusted *p* value < 0.1. In contrast, alterations in *TP53* and *PI3K* pathways were significantly enriched in CRC‐I, also with an adjusted *p* value < 0.1.^4^ The alterations in the WNT signaling pathway, which includes genes such as *APC* (67%), *SOX9* (13%), *CTNNB1* (11%), and *AXIN2* (9%), were observed in 80% of CRA patients. This prevalence suggested that WNT pathway activations were an early event in the development of CRA. The mutation rates within the RTK–RAS pathways were similar between CRA (53%) and CRC‐I (49%), with significant contributions from mutations in *KRAS* (39%), *BRAF* (5%), and *ERBB4* (5%). On the other hand, the *TP53* and *PI3K* signaling pathways were significantly more mutated in CRC‐1 compared with CRA (Fisher's exact test *p* ≤ 0.01). Specifically, the mutation rate in the TP53 signaling pathway was much lower in CRA (18%) than in CRC‐I (44%). Furthermore, the mutation rate in the PI3K pathway showed a threefold change from CRA (11%) to CRC‐I (31%), indicating its crucial involvement in the transition from precancerous lesions to carcinomas.

Meanwhile, the mutation spectrum of CRA has its unique significance in the early detection of CRC. We also analyzed another inheritable precancerous condition of CRC, familial adenomatous polyposis (FAP) and MAP (MUTYH‐Associated Polyposis).^35^, ^36^, ^37^ Compared with CRAs, we observed an increased mutation rate in *APC* (88 vs. 67%), a decreased mutation rate in *KRAS* (24 vs. 39%), and noted some mutations different from those in CRAs, such as *HLA‐DRB1* (26%), *MST1L* (14%), *FAM205A* (14%), *TAS2R43* (14%), and *PLCL1* (12%) (Figure 3D). Although CRA and FAP showed similar mutations in the WNT and RTK–RAS pathways, there were differences in the Hippo and PI3K pathways. The *TP53* pathway was found to be significantly mutated in CRA when compared with FAP/MAP (*p* value ≤ 0.01) (Figure 3E). This further indicates that CRAs possess their unique differential mutant genes, which hold certain research importance.

### 2.8 Cancer evolution analysis in CRA and CRC

**FIGURE 4:**
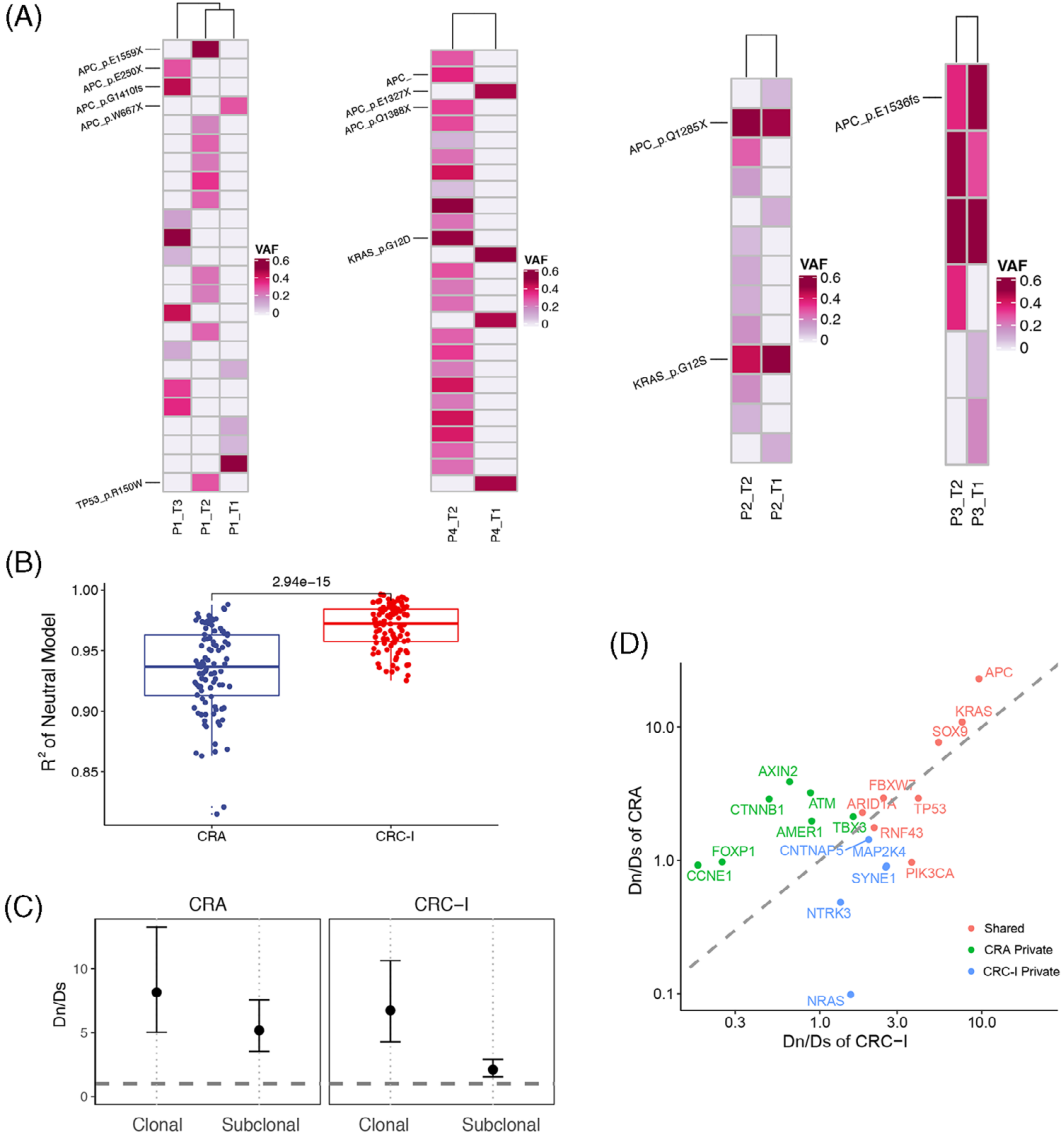
Evolutionary trajectories from CRA to CRC‐I. (A) The evolutionary paths of multiple adenomas. The oncoplots show the shared status of mutations across multiple adenomas. Somatic mutations of APC, KRAS and TP53 were labeled. (B) Neutral evolution index of CRA and CRC‐I. The squared correlation coefficient (R2) between the inverse allele frequency and the cumulative number of mutations is calculated. Tumors are considered to be neutrally evolved if R2 ≥ 0.98. (C) The temporal changes of Dn/Ds for clonal and subclonal truncating mutations in CRA and CRC‐I. (D) The scatter plot showing the Dn/Ds of driver genes in CRA and CRC‐I.

We delved into the evolutionary process from adenomas into carcinomas, by focusing on the emergence of multiple adenomas in individual patients. To shed light on the degree of intertumor heterogeneity among these adenomas, we analyzed four patients with multiple adenomas sequenced (Figure 4A). The analysis revealed two evolutionary routes for the development of these lesions, by either independent development or sharing a common ancestral origin. The mutational profiles in patients P1 and P4 had independent driver mutations for each lesion. Specifically, the first adenoma in patient P1 exhibited two mutations in the *APC* gene (E250X and G1410fs), the second had a unique *APC* mutation (E1559X) alongside a *TP53* mutation (R150W), and the third was characterized by a singular *APC* mutation (W667X). Contrastingly, adenomas exhibited shared mutations in Patients P2 and P3, indicating a potential common ancestral origin. Both adenomas in patient P2 harbored the same *APC* (Q1285X) and *KRAS* (G12S) mutations. Similarly, the adenomas in patient P3 shared three mutations, including one in *APC* gene (E1536fs). The diverse evolutionary origins of adenomas indicate distinct pathogenesis for neoplastic transformation.

In our investigation into the evolutionary dynamics underlying the transformation from CRA into CRC, we focused on the concept of clonal evolution, which encompasses both neutral and non‐neutral evolutionary processes.^38^ In tumors undergoing neutral evolution, driver mutations occur in the ancestral malignant cells and subsequent mutations had a neutral effect on tumor progression. Conversely, non‐neutral evolution is characterized by ongoing clonal selection and adaption, marked by the continuous acquisition of functional genomic alterations. Our analysis revealed that the neutral evolution index in CRC‐I was significantly higher than in CRA (Wilcoxon test, *p* = 2.94 × 10^−15^), indicating a predominant neutral evolution process in CRC‐I tumors, as opposed to the non‐neutral evolution observed in most CRA tumors (Figure 4B). The shift from non‐neutral into neutral evolution during the adenoma–carcinoma transition indicated that subclones in adenomas were under a non‐neutral scenario. As the tumor progresses, a dominant subclone overcomes growth constraints and outcompetes other subclones. This dominant subclone then accumulated genomic alterations in the late phase of tumor development, marking a shift into neutral evolution.^39^

Therefore, in examining the evolutionary dynamics within CRA and CRC‐I, we observed distinct patterns in the evolution of clonal (ancestral mutations) versus subclonal mutations (subsequent mutations). To quantify the selection strength within these tumors, we employed the Dn/Ds ratio test which compares the ratio of nonsynonymous to synonymous mutations. A higher Dn/Ds ratio corresponds to a stronger positive selection pressure (Figure 4C).^40^ For clonal mutations, both CRA and CRC‐I exhibited signs of positive selection, aligning with the expectations that most early genetic alterations significantly contribute to cancer development by driving tumorigenesis. Interestingly, when analyzing subclonal mutations, CRA displayed a notably higher selective pressure than CRC‐I. This suggested that subclones in CRA were subject to higher competition compared with those in CRC. This differential evolutionary pressure between CRA and CRC underscore the complex interplay of genetic mutations during the progression from precancerous lesions to cancer. Specifically, the observed dynamics suggest that while early alterations are universally crucial for tumorigenesis, the intensity of selection on subsequent mutations varies, reflecting a more competitive evolutionary landscape in CRA compared with CRC.

Furthermore, we estimated the selective strength of cancer genes in CRA and CRC‐I to pinpoint those that significantly influence tumorigenesis (Figure 4D). Genes experiencing positive selection (Dn/Ds ratio greater than 1) are identified as potential cancer driver genes.^41^ Despite mutation frequencies, eight genes were under comparable positive selection in both CRA and CRC‐I, including *APC*, *KRAS*, *SOX9*, *FBXW7*, *ARID1A*, *TP53*, *RNF43*, and *PIK3CA*, all of which are characterized as CRC driver genes in the IntOGen database.^25^ Meanwhile, five genes, including *CNTNAP5*, *MAP2K4*, *SYNE1*, *NTRK3*, and *NRAS*, were significantly mutated only in CRC‐I but not in CRA, indicating a specific role in CRC progression rather than in the early stages of CRA. Among these genes, *MAP2K4* and *NRAS* are reported as driver genes by IntOGen, while *NTRK3* is listed as a driver gene for esophageal cancer and stomach adenocarcinoma. *SYNE1* (Spectrin Repeat Containing Nuclear Envelope Protein 1) is involved in maintaining plasma membrane integrity and cytoskeletal structure, as well as in mitosis and cell cycle regulation. The overall mutation rate of *SYNE1* in TCGA CRC cohort (excluding MSI and *POLE*‐mutated samples) was 18%, with patients harboring *SYNE1* mutations exhibiting poorer overall survival (Log‐rank *p* = 0.0427). The role of *CNTNAP5* in CRC progression will be further elaborated in subsequent discussions.

### 2.9 A justified random forest model distinguishing CRA and CRC

**FIGURE 5:**
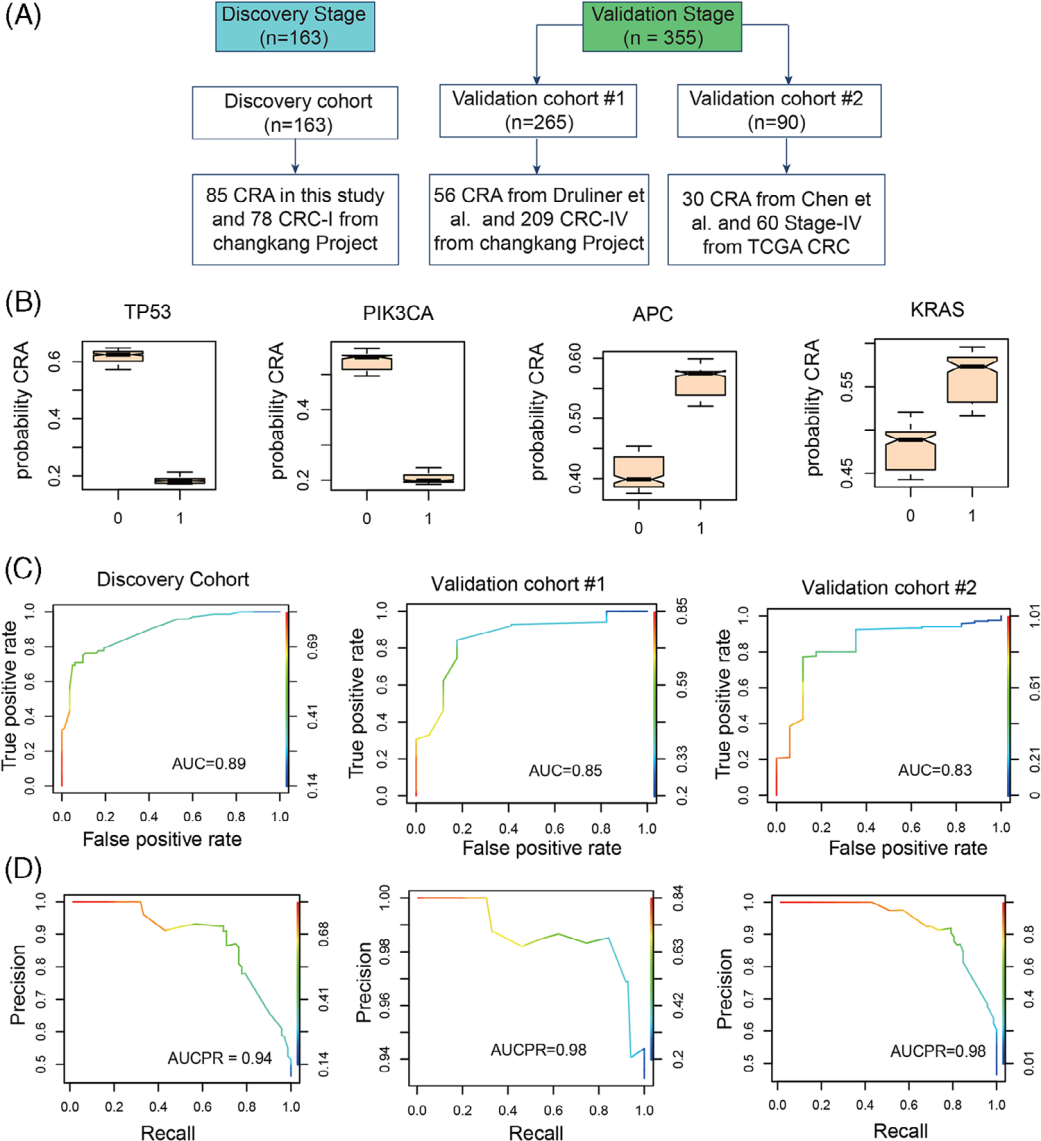
A random forest model for distinguishing malignant progression from CRA to CRC‐I. (A) Schematic illustration of the random forest model. (B) The marginal effect of genes on the class probability. The probability of CRA when the genes is wild‐type (*x* = 0) or mutant (*x* = 1). (C) The ROC curve (up) and precision–recall curve (Down) for the discovery cohort. ROC, receiver operating characteristic. (D and E) The model performance in the two validation cohorts.

To discern the genes that significantly distinguish CRA from CRC, we developed a random forest model using genes mutated in both CRA and CRC tissues (Figure 5A). The discovery dataset contained 163 patients, including 85 CRA in this study and 78 CRC‐I from the ChangKang Project. Two independent validation datasets were recruit. The validation cohort #1 contained 56 CRA from Druliner et al.^9^ and 209 CRC stage IV (CRC‐IV) from the ChangKang Project. The validation cohort #2 incorporated 90 patients, including 30 CRA patients from Chen et al.^42^ and 60 stage‐IV CRC patients from TCGA project. In discovery dataset, the random forest model identified 15 genes of significant importance, including *TP53, PIK3CA, KRAS, CNTNAP5, APC, GATA6, REL, AMER1, CTNNB1, LRP1B, AXIN2, FBXW7, ARID1A, NRAS*, and *ATM*. Among these genes, five genes, including *TP53, PIK3CA, CNTNAP5, FBXW7*, and *NRAS*, had higher mutation frequencies in CRC‐I compared with CRA, suggesting their pivotal roles in the progression of CRC (Figures 5B and S1). To further test the model's efficacy, we constructed a reduced random forest model utilizing these 15 crucial genes to categorize samples as either adenoma or CRC. The model achieved area under curves (AUC) of 0.89 in discovery dataset, with an out‐of‐bag error rate of 23% (Figure 5C). In validation datasets, the model demonstrated an AUC of 0.83–0.85, confirming the classifier stability and accuracy in differentiating between CRA and CRC (Figure 5D,E). The AUCPR (area under the precision–recall curve) values were 0.94 in the discovery dataset and 0.98 in the validation datasets, respectively, suggesting a good data balance in this model. Through the random forest model, we can screen genes with obvious mutational differences between CRC and CRA. By detecting the mutations of these genes, we can better understand the disease status and provide support for the early diagnosis of CRC.

### 2.10 Possible functions of newly identified cancer genes in CRC

In our comprehensive analysis aimed at identifying genes implicated in the malignant progression from CRA to CRC, *CNTNAP5* emerged as a gene of interest. *CNTNAP5* was found to be under positive selection in CRC compared with CRA, as indicated in our findings (Figure 4D), and it was also highlighted as a significant gene in the random forest model, displaying a higher mutation frequency in CRC than in CRA (Figure S1). *CNTNAP5* is known for its roles in cell adhesion and cellular communication within the nervous system and some studies have found its correlation with glaucoma.^43^ Further studies have found that *CNTNAP5* mutations can increase acetylated tubulin levels which may lead to cytoskeletal defects and disturbed cytoarchitecture. Meanwhile, *CNTNAP5* mutations may lead to greater apoptosis through increasing the level of cleaved caspase 3.^44^ However, its link to tumorigenesis has been minimally explored. Our analysis revealed that the majority of *CNTNAP5* mutations occur in the extracellular domain (Figure S2A). When examining the distribution of *CNTNAP5* mutations within the TCGA CRC dataset, a notable association was observed with *POLE*‐mutant (80%, eight out of 10 patients) and MSI subtypes (25.8%, 16 out of 62 patients), in contrast to genome stable (0%, none out of 58 patients) or copy number instability (3.4%, 11 out of 328 patients) (Figure S2B). To mitigate the effects of overall mutation burden as a confounding factor, our analysis specifically focused on *POLE*‐mutant and MSI subtypes. The TMB in *CNTNAP5*‐mutated samples was significantly higher than that of nonmutated samples (Wilcoxon test, *p* = 4.8 × 10^−4^) (Figure S2C), suggesting a potential association of *CNTNAP5* mutations with DNA repair pathways. Furthermore, we observed that patients with *CNTNAP5* mutations exhibited a marginally poorer disease‐free survival (DFS) than nonmutated samples (Log‐rank *p* = 0.088) (Figure S2D), underscoring the gene's potential significance in CRC carcinogenesis.

*GATA6*, identified as an important gene in our random forest model, exhibited a higher mutation rate in adenomas than in CRC. *GATA6*, as a member of the GATA family of zinc finger transcription factors, plays a crucial role in early extraembryonic development and the maintenance of tissue regenerative.^45^ Its involvement in CRC tumorigenesis is nuanced due to its dual functionalities within the WNT signaling pathway‐acting both as an activator (via *Lgr5*) or a repressor (via *BMP4*) of WNT target genes.^46^, ^47^ This dichotomy is reflected in the expression patterns of *GATA6*, which was elevated in CRC cells compared with normal colon cells (Figure S3A). Interestingly, a higher expression level of the *GATA6* gene is significantly associated with worse outcomes for patients, including both overall survival (Log‐rank *p *= 0.068) and DFS (Log‐rank *p *= 0.0093) (Figure S3B‐C). This suggests that while *GATA6* may contribute to the early stages of colorectal tumorigenesis, its increased expression in CRC is linked to adverse clinical outcomes.

## 3 DISCUSSION

The prognosis of CRC is heavily influenced by the tumor stage at diagnosis, underscoring the paramount importance of early screening and treatment for CRAs, which are precancerous lesions of CRC. Detection of DNA mutation genes in tissues provides us with direct insight into the nature of the lesions, which is of great significance for early disease diagnosis. Meanwhile, liquid biopsies, particularly the analysis of cfDNA, have garnered significant attention in detecting precancerous lesions in CRC, albeit with ongoing debates.^19^, ^20^, ^22^, ^23^ In our study, we focus on the value of cfDNA in precancerous lesions, instead of circulating tumor DNA (ctDNA).^48^ The ctDNA indicates DNA fragments that released into the blood circulation by tumor cells, which can reflect the tumor cell genetics to a certain extent and holds significant potential for tumor assessment, prognosis, and screening. However, the detection of ctDNA is hindered by low circulating content and a short half‐life. Moreover, ctDNA primarily mirrors the genomic alternations in tumor cells, limiting its application in early tumor monitoring and screening. On the other hand, cfDNA comprises free DNA fragments circulating in the blood. Research have shown that although the concentration of cfDNA in the blood of healthy people is low, it generally increases with cancer progression.^49^ Thus, understanding the changes in the genome of cfDNA not only enables the detection of tumor status but also provides vital guidance for precancer assessment and early screening.

In this study, we analyzed CRA tissues and matched plasma cfDNA from a cohort of 85 patients. Our findings reveal that a minute proportion of samples (five out of 85, 5.9%) successfully identified tissue‐reported variants within the matched cfDNA. To validate the mutation status in plasma, we found that gene mutations in plasma cfDNA can be detected by PCR and sequencing, which indicates the possibility for monitoring polyps’ status and detecting CRC at its very earliest, precarcinoma stage. Next, we explored potential biological factors affecting the detectability of cfDNA mutations in CRA. We observed that the genomic burden of CNVs in both tissue and plasma samples intensifies with malignancy, suggesting that CNV features could offer valuable information for the early detection applications of cfDNA. Recent studies have reported the utility of cfDNA fragment features in the early diagnosis of CRA and diverse cancers.^19^, ^20^ Further investigation into the clonal origins of CRA disclosed variations in clonal status among multiple CRAs within the same patient, including instances of independent origins and shared origins.^50^ Tumors emerging from a single founder tend to exhibit truncal alternations, which are more detectable in plasma samples.^22^ Additionally, the genetic landscape, particularly the co‐occurrence of *APC* and *KRAS* mutations, seems to influence the detectability of cfDNA mutations. CRAs harboring both *APC* and *KRAS* mutations may demonstrate a heightened predisposition toward carcinoma development. Regardless, identifying cfDNA mutations in precancerous lesions poses greater challenges than carcinomas. In a clinical context, enhancing detection sensitivity might involve screening patients with elevated risk factors. The findings of this study provide valuable insights for developing effective strategies for utilizing cfDNA in the early detection of precancerous lesions.

In this article, we have analyzed the differences in mutant genes between FAP and CRAs, as well as the related pathways which further illustrate the specific significance of studying CRAs. Additionally, this study delves into the genomic traits and the progression of cancer transformation from CRA to CRC‐I. By analyzing differences in mutation frequency, selection pressures, and gene significance within a random forest model, we have identified several genes, including *TP53, PIK3CA, CNTNAP5, FBXW7*, and *NRAS*, that have a higher mutation rate in CRC compared with CRA. These findings could potentially enhance the translational applications of a CRA–CRC classifier.^51^ Notably, *TP53* and *PIK3CA* emerged as dominant genes in this context. *PIK3CA*, a well‐established driver gene in CRC, is pivotal for cellular growth and proliferation through the PI3K/AKT signaling pathway.^34^ Besides, mutations in *FBXW7 and NRAS* have been associated with a diminished response to EGFR‐inhibitor therapy in metastatic CRC.^52^ In contrast, genes such as *APC*, *KRAS*, *CTNNB1*, and *GATA6*, showed a higher mutation frequency in precancerous lesions than in advanced cancer. Notably, the mutation rate of *CTNNB1* significantly decreased from CRA (11%) to CRC‐1 (1%), underscoring its role in activating canonical WNT signaling pathway. The high mutation frequency of *CTNNB1* in adenomas suggests its early driving function in CRA development, although oncogenic mutations in *CTNNB1* alone appear insufficient to initiate CRC tumorigenesis.^33^

This study faces several limitations that warrant further consideration. First, the small number of patients with AAs included in the study is due to the fact that early CRAs often lack overt symptoms, prompting patients to seek medical attention only when the disease advances to a more severe stage. Consequently, it is imperative to incorporate a larger patient cohort into the analysis. A primary challenge is the difficulty of conducting long‐term follow‐ups for patients with precancerous lesions under current conditions. Insights gained from existing long‐term follow‐up projects focused on precancerous lesions in lung,^12^ stomach,^53^ and other cancers highlight the importance of identifying key molecular markers for early cancer detection. Second, most samples in this study comprised early‐stage adenomas. In the future, collecting more samples of AAs may uncover new candidate makers for CRC early detection. Furthermore, the roles of newly identified genes, such as *CNTNAP5 and GATA6*, in CRC tumorigenesis remain to be fully elucidated. Future research should prioritize investigating the in vivo and in vitro functions of these genes to better understand their contribution to the development and progression of CRC.

## 4 MATERIALS AND METHODS

### 4.1 Patient recruitment

From December 2017 to January 2019, 85 participants were enrolled. Participants who underwent endoscopic polypectomy at SYSUCC were eligible if they were aged between 18 and 75 years and had at least one lesion histologically confirmed as polys, TAs, VAs, mixed TVAs, high‐risk adenomas, or cancer. When patients with multiple lesions, the advanced lesion was assigned as the finial pathological type. Blood and tissue samples from patients were collected for cancer gene‐targeted sequencing. The overall characteristics and long‐term outcomes of patients are summarized in Tables S1 and S2, respectively.

### 4.2 DNA extraction, library construction, and target sequencing

The DNA extraction and library construction processes were described in our previous studies,^16^, ^18^ Briefly, peripheral blood lymphocytes (serving as references) and plasma were separated from blood samples. DNA from lymphocytes was extracted using a RelaxGene Blood DNA System and cfDNA from plasma was extracted using the QIAamp Circulating Nucleic Acid Kit. For tissue samples, DNA was extracted from formalin‐fixed paraffin‐embedded (FFPE) using the QIAamp DNA FFPE tissue kit. The sequencing libraries for extracted DNA were prepared using the KAPA library preparation kit. Both tissue and plasma samples were sequenced using a specific panel targeting 451 cancer‐related genes, spanning 3.34 Mb. Sequencing was conducted with 150‐bp paired ends on the NovaSeq 6000 system.

### 4.3 CfDNA extraction, PCR, and sequencing

cfDNA from plasma samples were extracted according to the sample extraction method described above and were amplified with 2×T8 High‐Fidelity Master Mix as PCR templates, followed by sequencing and sequence alignment. Based on the gene sequencing results of cfDNA from plasma, we found the sequence of gene ZNF717 and specific primers were designed on NCBI (https://www.ncbi.nlm.nih.gov). The specific sequence diagram was analyzed and compared by the software Snapgene. The prime sequences were as following: rZNF717‐F: AAACCAATACCCATATTTATTTTTG, rZNF717‐R: GAAAAGCATGTTCTGGCAAT; ZNF717‐F: TTAAACCTTGGGATACACAAGAGA, ZNF717‐R: TCTCCCCCGTGTGAATACCTT.

### 4.4 Calling somatic mutations

Raw sequenced reads were preprocessed for quality controls by fastp v0.20.0. The cleaned reads were aligned to human references (hg19) using Burrows‐Wheeler Aligner (BWA) with the default setting.^54^ Duplicated reads were removed by Picard MarkDuplicates. After removing duplicated reads, the mean coverage was 2000× for blood control samples, 1000× for adenoma tissues, and 1500× for plasma samples. The somatic mutations were called by three different tools to obtain high confidence mutations: varscan2,^55^ strelka,^56^ and gvc.^57^ The mutations were further filtered by the following criteria.^58^ (1) AF of mutations in 1000 Genomes and ExAC ≤ 0.01. (2) Mapping ability ≥ 0.75. (3) The genomic distances between adjacent mutations were required ≥ 100 bp. For tissue samples, mutations detected by at least two tools were retained. For plasma samples, the mutations were identified by two additional criteria: (1) VAF of normal samples = 0; (2) VAF ≥ 0.05 if the mutation was detected by a single tool. All mutations were annotated using Annovar and processed into mutation annotation format (MAF).^59^

We also assessed whether somatic mutations in tissue samples could be detected from their matched plasma samples. Briefly, for somatic mutations detected from tissue samples, VAFs of mutations in matched cfDNA and leukocyte (control sample) were calculated using GetBaseCountsMultiSample (https://github.com/zengzheng123/GetBaseCountsMultiSample). Fisher's exact test was performed between the VAFs of cfDNA and control samples. We filtered for somatic mutations in cfDNA for the following criteria: (1) VAF in ctDNA ≥ 2 × VAF in normal; (2) *q* values of Fisher's exact test ≤ 0.1; (3) The number of alternative reads ≥ 4.

### 4.5 Detecting CNVs

The CNVs of tissue and plasma samples were detected by applying the FACETS copy number analysis tool.^29^ The number of references and alternative alleles read at germline single‐nucleotide polymorphisms (SNPs) in tumor and normal samples were calculated by the submodule snp‐pileup in FACETS at the parameters “‐q 15 ‐Q 20 ‐P100 ‐g ‐r20,0.” Then FACETS was run under insensitive mode for small CNVs by setting “cval = 150.” In the end, tumor purity, ploidy, allele‐specific CNVs, and cell fraction were determined by FACETS using the expectation‐maximization algorithm.

### 4.6 Identifications of genomic features of malignant progression from CRA into CRC‐I

The somatic mutations of CRC stage I (CRC‐I) patients were collected from the ChangKang Project.^4^ To coordinate whole exome sequencing in CRC‐I and target panel sequencing, somatic mutations of CRC‐I were restricted to the sequencing regions of panel sequencing in this study. The significantly mutated oncogenes and TSGs in the CRA were determined from nonsynonymous mutations using the SomInaClust R‐package software.^24^ Comprehensive genomic comparisons between CRA and CRC‐I were performed by R‐package maftools,^60^ including mutant frequency of driver genes, mutant residues of driver genes, and enrichment of known oncogenic signaling pathways.

The evolutionary processes from CRA to CRC‐I were also explored. The neutral evolution status of tumors was estimated using R‐package neutralitytestr (v.0.0.3).^38^ The subclonal mutations (0.1 < VAF < 0.25) of each sample were used to estimate whether tumors evolved neutrally or not. We used Dn/Ds to quantify the strength of selection for different sets of somatic mutations using R‐ package dndscv.^41^ The Dn/Ds was informative about the selection directions, where Dn/Ds > 1 if under positive selection (mutations promoting tumor's growth) and Dn/Ds < 1 if under negative selection (mutations impairing tumor's fitness).^40^, ^61^, ^62^ The somatic mutations were divided into clonal (VAF ≥ 0.25) and subclonal mutations (VAF < 0.25), where the cutoff was determined according to the overall VAF distributions. To detect significantly positively selected driver genes in CRA and CRC‐I, we calculate Dn/Ds at individual gene levels with the *p* value ≤ 0.05.

### 4.7 The FAP/MAP patient cohort

The somatic mutation of FAP/MAP cohort included three public studies, including Perne et al.,^35^ Rashid et al.,^63^ and Thomas et al.^37^ The raw mutations were downloaded and processed as CRC mutation described above. Mutations were limited to the 451‐gene panel in this study. In total, 15 FAP and 27 MAP patients, including 1,609 somatic mutations were collected for further analysis.

### 4.8 Construct a random forest model

A random forest model was built using R‐package random ForestSRC (v.2.13.0) on the datasets containing CRA and CRC‐I. The validation dataset consisted of colorectal adenoma mutations from a public study ^9^ and our CRC‐IV. Important variables were selected using the minimal depth variable selection method with the *p* value ≤ 0.05. A reduced model was constructed based on the identified important variables. The performance of the constructed model was estimated by the area under the receiver operating characteristic curve (abbreviated AUC) and the AUCPR on the training and validation datasets.

## AUTHOR CONTRIBUTIONS

*Conceived the project, conceptualization, supervision, project administration, and funding acquisition*: L. H. Y. and X. R. H. *Data curation, software, formal analysis, investigation, visualization, and methodology*: C. Q. J. and X. Y. H. *Pathological analysis, resource, data curation, investigation, and methodology*: K. S. Y., L. W. H., L. L. N., and Y. L. P. *Investigation and methodology*: Z. Q. H., Y. P., H. J. Q., Z. X. N., Z. J., and Z. Q. *Writing the original draft, writing—review and editing*: L. H. Y., X. R. H., C. Q. J., X. Y. H., L. W. H., L. L. N., and K. S. Y. All the authors reviewed and approved the manuscript.

## CONFLICT OF INTEREST STATEMENT

Authors Yang Pan, Xiaoni Zhang and Jing Zhang are employees in HaploX Biotechnology but have no potential relevant financial or nonfinancial interests to disclose. The other authors have no conflicts of interest to declare.

## ETHICS STATEMENT

This retrospective study was designed and approved by the Sun Yat‐Sen University Cancer Center (SYSUCC) Ethics Committee (B2019‐031‐01, Guangdong, China).

## Supporting information



Supporting information

## Data Availability

The raw DNA sequencing data in this paper have been uploaded into the Genome Sequence Archive in National Genomics Data Center, China National Center for Bioinformation/Beijing Institute of Genomics, Chinese Academy of Sciences, under the project accession number: PRJCA026333 (https://ngdc.cncb.ac.cn/gsa‐human/) with restricted assess. Raw data request can be obtained by completing the application form via the CNCB‐NGDC system.
