# Supplementary material for: The tissue and circulating cell‐free DNA‐derived genetic landscape of premalignant colorectal lesions and its application for early diagnosis of colorectal cancer
Source: MedComm (2020). 2024 Nov 14;5(12):e70011. doi: 10.1002/mco2.70011 (PMC11564342; doi:10.1002/mco2.70011)
Supplement: Supplementary file 1 — Supporting information [file MCO2-5-e70011-s001.docx]

**The tissue and circulating cell-free DNA-derived genetic landscape of pre-malignant colorectal lesions and its application for early diagnosis of colorectal cancer**

Qingjian Chen^1,2,4#^, Yu-Hong Xu^1,2#^, Shiyang Kang^3#^, WuHao Lin^1,2#^, Linna Luo^2,5#^, Luping Yang^1,2^, Qi-Hua Zhang^1,2^, Ya Pan^6^, Jia-Qian Huang^1,2^, Xiaoni Zhang^6^, Jing Zhang^6^, Qi Zhao^1,2^, Rui-Hua Xu^1,2^*, Hui-Yan Luo^1,2^*

1. Department of Medical Oncology, Sun Yat Sen University Cancer Center, State Key Laboratory of Oncology in South China, Collaborative Innovation Center for Cancer Medicine, Sun Yat-sen University, Guangzhou 510060, P. R. China.

2. Research Unit of Precision Diagnosis and Treatment for Gastrointestinal Cancer, Chinese Academy of Medical Sciences, Guangzhou 510060, P. R. China.

3. Department of Anaesthesiology, Sun Yat Sen University Cancer Center, State Key Laboratory of Oncology in South China, Collaborative Innovation Center for Cancer Medicine, Sun Yat-sen University, Guangzhou 510060, P. R. China.

4. State Key Laboratory of Systems Medicine for Cancer, Department of Oncology, Shanghai General Hospital, Shanghai Jiao Tong University School of Medicine, Shanghai 200080, P.R. China

5. Department of Endoscopy, Sun Yat Sen University Cancer Center, State Key Laboratory of Oncology in South China, Collaborative Innovation Center for Cancer Medicine, Sun Yat-sen University, Guangzhou 510060, P. R. China

6. HaploX Biotechnology, Shenzhen 518057, P. R. China.

^#^ Qingjian Chen, Yu-Hong Xu, Shiyang Kang, WuHao Lin, Linna Luo contributed equally.

^*^ Correspondence to:

Hui-Yan Luo: [luohy@sysucc.org.cn](mailto:luohy@sysucc.org.cn), Rui-Hua Xu: [xurh@sysucc.org.cn](mailto:xurh@sysucc.org.cn)

**Supplementary Figures and Tables**

**Table S1: The overall patient characteristics**

| Characteristics | Polyps | TAs | TVAs | High-Risk | Cancer |
| --- | --- | --- | --- | --- | --- |
| Total | 3 | 10 | 52 | 14 | 6 |
| Gender |  |  |  |  |  |
| Male, 50 (58.8%) | 3 | 6 | 31 | 8 | 2 |
| Female, 35 (41.2%) | 0 | 4 | 21 | 6 | 4 |
| Age |  |  |  |  |  |
| ＜ 61, 43 (50.6%) | 3 | 5 | 29 | 4 | 2 |
| ＞= 61, 42 (49.4%) | 0 | 5 | 23 | 10 | 4 |
| Location |  |  |  |  |  |
| Left, 48 (56.5%) | 2 | 4 | 30 | 8 | 4 |
| Right, 22 (25.9%) | 1 | 5 | 12 | 4 | / |
| Unknown, 15 (17.7%) | / | 1 | 10 | 2 | 2 |
| Number |  |  |  |  |  |
| Single, 17 (20.0%) | 1 | 5 | 9 | 2 | 0 |
| Multiple, 68 (80.0%) | 2 | 5 | 43 | 12 | 6 |

**Table S2: The long-term outcomes of patients**

| **Follow-up** | **1 year** | **2 years** | **3 years** | **4 years** | **5 years** | **6 years** |
| --- | --- | --- | --- | --- | --- | --- |
| **Total** | 16 | 12 | 20 | 6 | 8 | 1 |
| **Gender** |  |  |  |  |  |  |
| Male | 10 | 8 | 12 | 5 | 5 | 1 |
| Female | 6 | 4 | 8 | 1 | 3 | 0 |
| **Polyps** |  |  |  |  |  |  |
| 0 | 3 | 6 | 8 | 4 | 4 | 0 |
| ＞0 | 13 | 6 | 10 | 2 | 4 | 1 |
| **Cancer** | 0 | 0 | 2 | 0 | 0 | 0 |

**Table S3: Summary of ZNF717 mutations in 6 plasma samples.**

| **sample** | **Reference_Allele** | **Variant_Type** | **Result** | **Mutation type** |
| --- | --- | --- | --- | --- |
| 1 | T | SNP | T/T | Wild type |
| 2 | T | SNP | T/T | Wild type |
| 3 | T | SNP | T/T | Wild type |
| 4 | G | SNP | G/A | Heterozygous mutation |
| 5 | G | SNP | A/A | Homozygous mutation |
| 6 | A | SNP | A/A | Wild type |

**Figure S1: The marginal effect of genes on the class probability for 15 selected genes.** The probability of CRA when the genes is wild-type (x = 0) or mutant (x = 1).

**Figure S2: Functional effects of CNTNAP5 in TCGA CRC**. (A) Mutated sites are shown for CNTNAP5 mutations in TCGA CRC. (B) Proportion of mutated and wild-type CNTNAP5 samples in TCGA CRC subtypes. (C) TMB of mutated and wild-type CNTNAP5 samples in TCGA CRC (POLE and MSI subtypes only). (D) The disease-free ratio of mutated and wild-type CNTNAP5 samples in TCGA CRC (POLE and MSI subtypes only).

**Figure S3: Functional effects of GATA6 in TCGA CRC**. (A) The expression levels of GATA6 gene in tumor (red) and normal tissues (grey). (B-C) The overall survival (OS) and disease-free survival (DFS) for patients with high (red) and low GATA6 (blue) expression.
